# Short- and Long-Term Hearing Outcomes After Hyperbaric Oxygen Therapy in Idiopathic Sudden Sensorineural Hearing Loss

**DOI:** 10.5152/eurasianjmed.2026.261486

**Published:** 2026-06-30

**Authors:** Ozan Kuduban, Recep Özkan

**Affiliations:** 1 Department of Otolaryngology, Head and Neck Surgery, Atatürk University, Erzurum, Türkiye; 2 Department of Undersea and Hyperbaric Medicine, Atatürk University, Erzurum, Türkiye

**Keywords:** Audiometry, hyperbaric oxygen therapy, prognosis, sudden sensorineural hearing loss, treatment outcome

## Abstract

**Background:**

Sudden sensorineural hearing loss (SSNHL) is an otologic emergency with a highly variable clinical course. Hyperbaric oxygen therapy (HBOT) has been widely used as an adjunctive treatment; however, its efficacy and determinants of treatment response remain incompletely defined. The aim of this study was to evaluate hearing outcomes in patients receiving HBOT and to assess the association between treatment-related factors and audiometric recovery.

**Methods:**

This retrospective study included 65 patients with idiopathic SSNHL. Pure tone audiometry thresholds were evaluated at baseline, post-treatment, and long-term follow-up when available. Changes were analyzed with the Wilcoxon signed-rank and Friedman tests, and correlations with recovery were assessed using Spearman’s analysis.

**Results:**

Complete pre- and post-treatment data were available for 65 patients. A statistically significant improvement in hearing thresholds was observed, decreasing from 41.7 ± 21.9 dB at baseline to 31.9 ± 22.1 dB following HBOT (*P* < .001). Among 19 patients with long-term follow-up, hearing thresholds further improved to 25.9 ± 17.3 dB. A significant difference was observed between baseline and long-term measurements (*P* = 0.006), although the overall comparison did not reach statistical significance (*P* = .209). No significant correlations were identified between hearing improvement and the number of HBOT sessions (*r* = 0.11, *P* = .387) or treatment delay (*r* = −0.06, *P* = .658). Etiological factors observed in the study population included upper respiratory infection (n = 30), acoustic barotrauma (n = 2), trauma (n = 1), and cases with no identifiable cause (n = 32). When analyzed according to etiology, no statistically significant difference in hearing threshold improvement was observed between groups (Kruskal–Wallis test, *P* = .151).

**Conclusion:**

HBOT demonstrated a positive effect on hearing thresholds in patients with SSNHL, with the observed audiological improvements appearing to be sustained at long-term follow-up.

**Main Points**

- Hyperbaric oxygen therapy (HBOT) was associated with short-term improvement in hearing thresholds in patients with idiopathic sudden sensorineural hearing loss (SSNHL).
- Long-term follow-up data suggested that hearing improvement may persist beyond the treatment period.
- HBOT may be considered a valuable adjunctive option in the management of idiopathic SSNHL.

## Introduction

Sudden sensorineural hearing loss (SSNHL) is defined as a hearing loss of at least 30 decibels (dB) affecting 3 or more consecutive frequencies within 72 hours without an identifiable cause.^1^ Despite its relatively low incidence, SSNHL constitutes an otolaryngologic emergency due to its abrupt onset and the potential for permanent hearing impairment if treatment is delayed.^2^ The underlying pathophysiology remains incompletely understood; proposed mechanisms include viral infections, vascular compromise, and immune-mediated processes.^2^^,^^3^

The clinical course of SSNHL is highly variable, with spontaneous recovery reported in a subset of patients. Nevertheless, a substantial proportion experience persistent hearing loss, resulting in significant functional limitations and psychosocial burden. Several prognostic factors have been implicated in recovery, including age, initial severity of hearing loss, presence of vertigo, and time to treatment initiation.^3^

Corticosteroids remain the mainstay of therapy and may be administered systemically or via intratympanic injection.^1^^,^^2^ In recent years, hyperbaric oxygen therapy (HBOT) has emerged as an adjunctive or salvage treatment modality.^4^ The rationale for HBOT lies in its ability to enhance oxygen delivery to the cochlea, thereby improving metabolic activity and promoting recovery in hypoxic inner ear tissues.^5^^,^^6^

Despite growing utilization of HBOT in SSNHL management, its clinical efficacy and the determinants of treatment response remain controversial.^6^^,^^7^ In particular, the influence of treatment timing, number of HBOT sessions, and patient-specific factors on audiometric outcomes has not been fully elucidated.

Accordingly, the present study aimed to evaluate the effect of HBOT on hearing outcomes in patients with sudden idiopathic SNHL and to investigate potential factors associated with treatment response, including treatment delay and the number of HBOT sessions.

## Material and Methods

### Study Design and Population

This retrospective observational study comprised 65 patients diagnosed with sudden idiopathic SNHL (AISNHL) who underwent HBOT at Atatürk University Research Hospital between 2023 and 2025. Patients with incomplete audiometric threshold data, including missing pure tone audiometry (PTA) or air- and bone-conduction measurements required for analysis, were excluded. Long-term follow-up data were available for a subset of the cohort. Treatment delay was defined as the time interval (in days) between the onset of symptoms and hospital presentation. This study used ChatGPT only for language editing and grammar improvement during manuscript preparation. All content was critically reviewed, revised, and approved by the authors.

### Ethical Approval

This retrospective study was approved by the Non-Interventional Clinical Research Ethics Committee of Atatürk University (Meeting No: 11; Decision No: 27; Date: December 26, 2025). The study was conducted in accordance with the Declaration of Helsinki. The requirement for written informed consent was waived by the local ethics committee because of the retrospective nature of the study and the use of anonymized patient data.

### Treatment Protocol

All patients received HBOT according to institutional protocols. Treatment was administered in a multiplace hyperbaric chamber at approximately 2.4 ATA with 100% oxygen. The number of HBOT sessions varied depending on clinical response and physician discretion. All patients received standard-of-care treatment for SSNHL, including systemic and/or intratympanic corticosteroid therapy, in addition to HBOT.

### Audiometric Assessment

Pure tone audiometry data were retrospectively obtained from medical records at baseline (prior to HBOT), after completion of HBOT, and at long-term follow-up when available. Post-treatment audiometric assessments were based on routine clinical evaluations and were not performed according to a standardized protocol. Long-term follow-up data were included when available in the hospital records, and no additional follow-up assessments were scheduled as part of the study. Hearing threshold levels were calculated as the average hearing threshold at standard frequencies (500, 1000, 2000, and 4000 Hz). Lower hearing threshold levels (PTA values) indicate better hearing function. Audiometric evaluations were performed at variable time points across the patient cohort.

### Outcome Measures

Clinical outcomes were categorized based on changes in PTA values as follows: complete recovery (final PTA ≤ 25 dB), partial recovery (≥10 dB improvement), and no recovery (<10 dB improvement).

### Statistical Analysis

Continuous variables are expressed as mean ± standard deviation (SD) or median (interquartile range), as appropriate. Normality of data distribution was assessed using the Kolmogorov–Smirnov test. For paired comparisons between baseline and post-treatment measurements, the Wilcoxon signed-rank test was used. For comparisons across 3 time points (baseline, post-treatment, and long-term follow-up), the Friedman test was applied. Correlation analyses between clinical variables, including HBOT session number and treatment delay, and audiometric improvement were performed using Spearman’s rank correlation coefficient. Statistical analyses were performed using IBM SPSS Statistics for Windows, Version 26.0 (IBM Corp., Armonk, NY, USA). A *P *value < .05 was considered statistically significant.

## Results

A total of 65 patients with complete audiometric data were included in the final analysis. The mean age was 44.7 ± 16.4 years (range: 18–74). The cohort consisted of 33 females (50.8%) and 32 males (49.2%). The affected ear was right in 26 patients (40.0%), left in 20 patients (30.8%), and bilateral in 19 patients (29.2%) (Table 1).

The number of HBOT sessions ranged from 5 to 30, with a mean of 13.2 ± 6.5 sessions, reflecting individualized treatment decisions based on clinical response.

**Figure 1.**
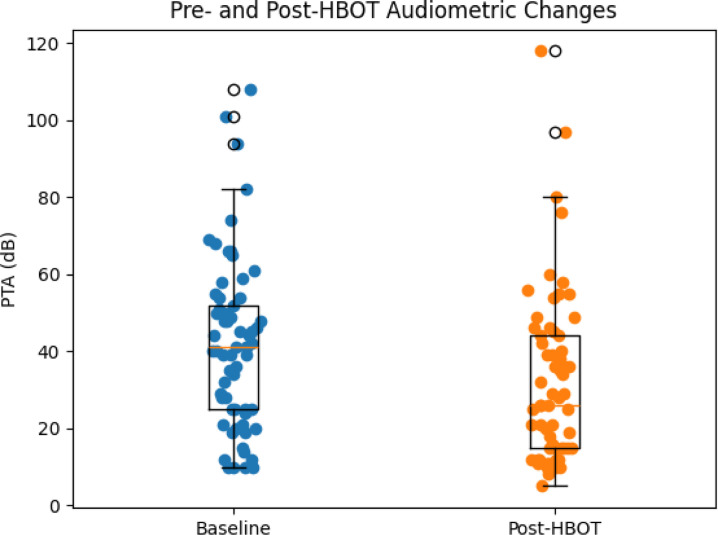
Distribution of pure tone average (PTA) values before and after hyperbaric oxygen therapy (HBOT). Boxplots represent median and interquartile range, while individual data points are overlaid.

A total of 65 patients had complete pre- and post-treatment audiometric data. Hearing threshold levels decreased from 41.7 ± 21.9 dB at baseline to 31.9 ± 22.1 dB after HBOT, indicating a significant improvement (Wilcoxon test, *P* < .001) (Table 2, Figure 1).

**Figure 2.**
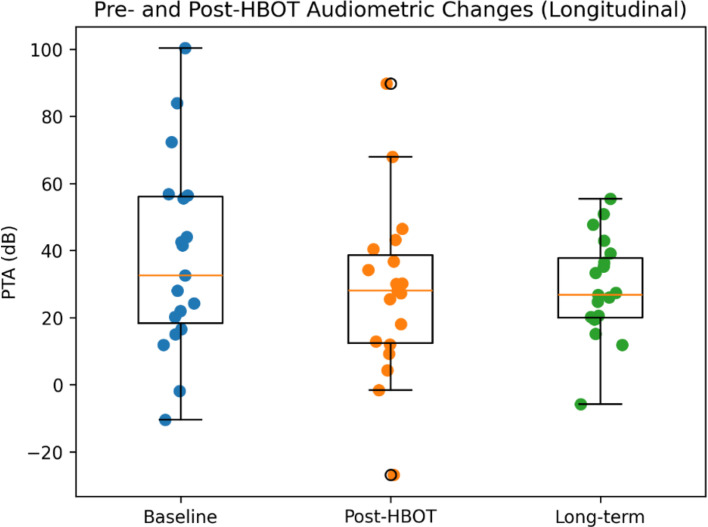
Distribution of pure tone average (PTA) values at baseline, after HBOT, and at long-term follow-up. Boxplots represent data distribution, individual data points are overlaid, and the mean trend is illustrated across time points.

Nineteen patients had complete audiometric data available at all time points. Hearing threshold levels decreased from 43.2 ± 24.7 dB at baseline to 34.3 ± 25.6 dB after HBOT and further improved to 25.9 ± 17.3 dB at long-term follow-up (Table 3, Figure 2). Although the overall comparison did not reach statistical significance (Friedman test, *P* = .209), a significant improvement was observed between baseline and long-term measurements (*P* = .006) (Table 4). This discordance can be explained by the limited sample size and reduced statistical power of the overall test, as well as variability across time points, which can result in significant pairwise differences despite a non-significant global test.

Clinical outcomes following HBOT are presented in Table 5. Complete recovery was observed in 46.2% of patients, while 7.7% showed partial recovery. No improvement was observed in 46.2% of patients.

The mean treatment delay was 12.8 ± 7.9 days, ranging from 2 to 30 days between symptom onset and hospital admission. Correlation analysis revealed no significant association between the number of HBOT sessions and audiometric improvement (Spearman *r* = 0.11, *P* = .387). Similarly, treatment delay was not associated with hearing recovery (Spearman* r* = −0.06, *P* = .658) (Table 6).

When analyzed according to etiology, no statistically significant difference in audiometric improvement was observed between groups (Kruskal–Wallis test, *P* = .151). However, patients with upper respiratory infection–related hearing loss showed a trend toward greater improvement compared to other groups.

## Discussion

This study suggests that HBOT is associated with significant improvement in hearing thresholds in patients with SSNHL, with effects that appear to extend into the long term. Notably, neither treatment delay nor the number of HBOT sessions showed a significant association with audiometric recovery, suggesting that treatment response may not be solely driven by timing or treatment intensity. Spontaneous recovery is a well-recognized phenomenon in SSNHL, reported in a substantial proportion of patients, which complicates the interpretation of treatment-related effects in uncontrolled studies.^8^^,^^9^

The observed reduction in hearing threshold levels following HBOT corroborates accumulating evidence that enhanced oxygen delivery to the cochlea mitigates hypoxia-related metabolic dysfunction in inner ear tissues.^4^^-^^6^ Consistent with prior clinical studies and meta-analyses, the findings reinforce the role of HBOT as a commonly used adjunctive or salvage therapy in SSNHL.^7^^10-13^

The magnitude of improvement in hearing thresholds observed in the cohort further supports its clinical utility, particularly in the context of multimodal treatment strategies.^12^ ^13^

A key finding of this study is the persistence and potential progression of hearing improvement at long-term follow-up. Although some comparisons did not achieve statistical significance, the persistent difference from baseline supports the possibility that HBOT-related improvements may be maintained over time. This observation is clinically relevant and supports a paradigm in which therapeutic benefits extend beyond the immediate post-treatment period.

Contrary to prevailing assumptions, treatment delay was not significantly associated with hearing recovery in the cohort. Previous studies have suggested that treatment delay may influence outcomes, with earlier initiation associated with better hearing recovery.^8^ This finding diverges from prior reports emphasizing early intervention as a critical determinant of outcome. However, this discrepancy likely reflects methodological differences, particularly in the definition of treatment delay. In the present study, delay was defined as the interval to hospital presentation rather than the initiation of HBOT, which may have attenuated detectable associations. Additionally, limited statistical power may have contributed to this negative finding.

Similarly, the absence of a dose–response relationship between the number of HBOT sessions and audiometric improvement challenges the assumption that greater treatment exposure necessarily translates into superior outcomes. Instead, the data suggest that intrinsic patient-related factors may play a more dominant role in determining recovery trajectories. This interpretation aligns with prior evidence highlighting the prognostic significance of baseline severity, age, and comorbid conditions.^14^

Subgroup analyses revealed a trend toward greater improvement among patients with upper respiratory infection–associated SSNHL; however, this did not reach statistical significance. While limited by small subgroup sizes, this finding raises the possibility of etiologically driven variability in treatment responsiveness and warrants further investigation. Several limitations should be considered. The retrospective nature of the study introduces the potential for selection bias, while the relatively small sample size—particularly in subgroup and long-term analyses—may limit the generalizability of the findings. In particular, the relatively small number of patients with long-term follow-up (n = 19) further limits the generalizability of long-term outcomes. A key limitation of this study is the absence of a control group, which substantially limits the ability to draw causal inferences regarding the effect of HBOT on hearing outcomes. In the absence of a control group, observed improvements cannot be definitively attributed to HBOT and may reflect the natural course of the disease or spontaneous recovery. Therefore, the findings of the present study should be interpreted with caution, particularly in light of the retrospective design, absence of a control group, and the potential contribution of spontaneous recovery.

Despite these limitations, this study provides real-world observational data suggesting that HBOT is associated with improvements in hearing outcomes in patients with SSNHL. The inclusion of longitudinal follow-up enhances the clinical relevance of the findings, with improvements appearing to persist over time. These findings should be considered in the context of existing controlled studies, and further prospective studies may help to refine patient selection, treatment timing, and optimal protocols.

## Figures and Tables

**Table 1.** Demographic and Clinical Characteristics of the Study Population

Variable	n (%) / Mean ± SD
Total patients	65
Age (mean ± SD)	44.7 ± 16.4 years
Age range (years)	18-74
Female	33 (50.8)
Male	32 (49.2)
Affected ear	
Right	26 (40)
Left	20 (30.8)
Bilateral	19 (29.2)

Data are presented as number (percentage) for categorical variables and mean ± standard deviation (SD) for continuous variables.

**Table 2.** Comparison of Pure Tone Average (PTA) Values Before and After Hyperbaric Oxygen Therapy (HBOT)

Group	n	Mean ± SD (dB)	Median	*P*
Baseline	65	41.7 ± 21.9	41	
Post-HBOT	65	31.9 ± 22.1	26	<.001

Only patients with complete paired audiometric data were included in the analysis.

**Table 3.** Changes in Pure Tone Average (PTA) Values in Patients with Available Long-term Follow-up Data

Group	n	Mean ± SD (dB)	Median
Baseline	19	43.2 ± 24.7	41
Post-HBOT	19	34.3 ± 25.6	29
Long-term	19	25.9 ± 17.3	26

Measurements were compared at baseline, after hyperbaric oxygen therapy (HBOT), and at long-term follow-up.

**Table 4.** Statistical Comparison of Pure Tone Average (PTA) Values Across Different Time Points

Comparison	*P*
Baseline vs. Post-HBOT	.286
Post-HBOT vs. Long-term	.236
Baseline vs. Long-term	**.006**
Overall ctomparison	.209

*P* values represent comparisons between baseline, post-treatment, and long-term measurements in patients with complete data. Overall comparison was performed using a non-parametric test, and pairwise comparisons were conducted between time points.

Long-term follow-up PTA values were significantly lower than baseline values (p=0.006), suggesting sustained hearing improvement. However, the overall Friedman test was not significant (p=0.209), and therefore this finding should be interpreted cautiously given the small sample size (n=19).

**Table 5.** Distribution of Clinical Outcomes Based on Changes in Pure Tone Average (PTA) Values Following Hyperbaric Oxygen Therapy (HBOT)

Outcome	Definition	n (%)
Complete recovery	Final PTA ≤25 dB	30 (46.2)
Partial recovery	≥10 dB improvement	5 (7.7)
No recovery	<10 dB improvement	30 (46.2)

Outcomes were categorized as complete recovery, partial recovery, or no recovery according to predefined criteria.

**Table 6.** Correlation Analysis Between Clinical Variables and Audiometric Improvement

Variable	Outcome	*r*	*P*
HBOT sessions	PTA improvement	0.11	.387
Treatment delay	PTA improvement	−0.06	.658

Spearman correlation coefficients (*r*) were calculated to assess the relationship between the number of hyperbaric oxygen therapy (HBOT) sessions, treatment delay, and changes in pure tone average (PTA). Audiometric improvement was defined as the difference between baseline and post-treatment PTA values.

## Data Availability

The data that support the findings of this study are available on request from the corresponding author.
